# Racetrack memory based on in-plane-field controlled domain-wall pinning

**DOI:** 10.1038/s41598-017-00837-x

**Published:** 2017-04-11

**Authors:** Fanny Ummelen, Henk Swagten, Bert Koopmans

**Affiliations:** Department of Applied Physics, Eindhoven University of Technology, P.O. Box 513, 5600 MB, Eindhoven, The Netherlands

## Abstract

Magnetic domain wall motion could be the key to the next generation of data storage devices, shift registers without mechanically moving parts. Various concepts of such so-called ‘racetrack memories’ have been developed, but they are usually plagued by the need for high current densities or complex geometrical requirements. We introduce a new device concept, based on the interfacial Dzyaloshinskii-Moriya interaction (DMI), of which the importance in magnetic thin films was recently discovered. In this device the domain walls are moved solely by magnetic fields. Unidirectionality is created utilizing the recent observation that the strength with which a domain wall is pinned at an anisotropy barrier depends on the direction of the in-plane field due to the chiral nature of DMI. We demonstrate proof-of-principle experiments to verify that unidirectional domain-wall motion is achieved and investigate several material stacks for this novel device including a detailed analysis of device performance for consecutive pinning and depinning processes.

## Introduction

Driven by the ever increasing demand for denser and faster data storage media, novel memory devices are being explored by the spintronics community. One of these novel devices is the so-called racetrack memory, a magnetic strip in which information is stored as magnetic domains that can be transported along the strip^1^, without the requirement of any mechanically moving components. Over the years, various versions of this device have been developed; made of in-plane or out-of-plane magnetic strips^2, 3^, based on the spin transfer torque or on spin-orbit torque^4, 5^ and in single layers or in exchange coupled stacks^6^. What is a common factor in all these racetrack versions is that an electrical current needs to run through the strip in order to move the domains, or equivalently the domain walls. A racetrack based on domain wall motion driven by magnetic fields is considered unfeasible, because these fields drive up-down and down-up domain walls in opposite direction, resulting in annihilation of the stored information. This is unfortunate, because field driven devices posses some beneficial properties: they are unhindered by Joule heating, which poses a major problem when driving large currents through small wires^7^, electrical contacts are not required, when designed cleverly power consumption might be small^8^, and their lifetime is not limited by electromigration^9^.

Over the last decade, several creative approaches to circumvent this seemingly unsurmountable problem have been put forward. One of them is a domain wall ratchet created by a saw tooth shaped anisotropy profile or asymmetric notches^10, 11^. Drawbacks of such ratchet compared to the conventional racetrack are that the information can only be moved in one direction, and the complex structural modulation makes it unlikely to be implemented in industry. Another interesting idea is to make use of the precession torque that a magnetic field exerts on the magnetic moments inside the domain wall^12, 13^. However, this mechanism of domain wall motion has not yet been demonstrated in materials with perpendicular magnetic anisotropy (PMA).

Another approach is the so-called bubblecade memory^14^. Recently it was observed that field-induced growth of reversed domains becomes asymmetrical in the presence of in-plane magnetic fields^15–17^. Based on these experiments it was shown that magnetic bubbles could be moved unidirectionally by expanding and shrinking them asymmetrically. The underlying physical phenomenon is the Dzyaloshinskii-Moriya interaction (DMI), which stabilizes chiral Néel walls, leading to a difference in DW energy (and hence a different DW velocity) when parallel or antiparallel in-plane magnetic fields are applied. This antisymmetric type of exchange interaction is intensively researched because of its importance for spin-orbit torque driven domain wall motion^5, 18^ and for the formation of magnetic skyrmions^19, 20^.

In this work, an alternative physical design is proposed for a purely magnetic-field-driven racetrack memory. We were inspired by the recent observation that a combination of an in-plane magnetic field and interfacial DMI causes a significant asymmetry in the domain-wall depinning field at an anisotropy barrier^21^. Based on this particular phenomenon, we have designed magnetic tracks with an effective interfacial DMI, combined with a lateral modulation of the perpendicular magnetic anisotropy using local ion irradiation. It is demonstrated that unidirectional motion of multiple domains walls is achieved for alternating in-plane and out-of-plane field combinations, fully in line with the underlying physics of DMI-induced depinning asymmetry. In the following, experimental data proving the device concept will be gathered emphasizing the decisive role of the direction of the additional symmetry-breaking in-plane magnetic field. Moreover, several material combinations to explore different strengths of DMI will be explored, yielding significant, sometimes unexpected changes in effective domain-wall movement. Our data include a careful analysis of the success rate of the observed unidirectional motion. This is an important step towards a further understanding of the physics processes and, though many technical challenges remain regarding scaling down and the control of orthogonal magnetic fields, the potential implementation in future memory devices.

## Results

### Device concept

**Figure 1:**
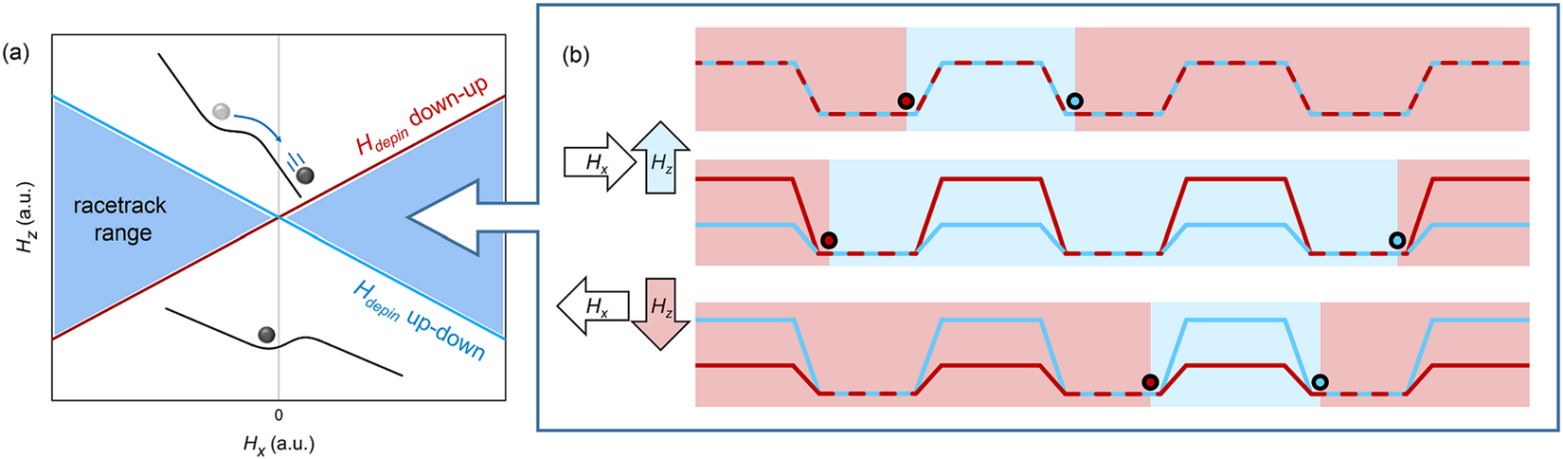
Device concept. (**a**) Schematic graph of the change in *H*_*depin*_ (both for up-down and down-up domain walls) as a function of *H*_*x*_. If *H*_*z*_ is below *H*_*depin*_ a DW stays pinned at an anisotropy barrier while above *H*_*depin*_ it will move past it, as indicated by the black inset cartoons. (**b**) An initial magnetic configuration is shown in the top cartoon (red shading = down, blue shading = up) together with a schematic energy landscape for the domain walls, which are represented by circles. *H*_*x*_ lowers the energy barriers for one type of DW, which is subsequently moved by an *H*_*z*_ pulse, and the system ends up in the configuration shown in the middle cartoon. A following *H*_*x*_ and *H*_*z*_ with opposite sign move the other type of DWs in the same direction, ending up in the configuration shown in the bottom cartoon.

In systems with PMA, a step in the magnetic anisotropy forms a pinning site for domain walls^22^. The magnetic field directed perpendicular to the sample plane (z direction) necessary to overcome such energy barrier, *H*_*depin*_, is determined by the difference in domain wall energy in the low and high anisotropy regions and by the width of the barrier. Recently it was demonstrated that an additional in-plane magnetic field, *H*_*x*_, influences *H*_*depin*_ in systems with DMI^21^. Because of the chiral nature of DMI, the change in *H*_*depin*_ is different for up-to-down (UD) and down-to-up (DU) domain walls. Figure 1(a) schematically shows this dependence for the two types of domain walls (although in actual experiments the behaviour will be more complex). It can be seen that for a certain value of *H*_*x*_ there exist a range of *H*_*z*_ (the blue region in Fig. 1(a)) resulting in depinning of UD walls but not of DU walls (or vice versa, depending on the sign of the DMI). Because of the symmetry of the situation, at −*H*_*x*_ DU walls will now move while UD walls remain pinned for the same range of *H*_*z*_. Domain wall motion driven by *H*_*z*_ will always be such that either the up or the down domains grow, which implies that UD and DU walls are driven in opposite directions. Utilizing the influence of *H*_*x*_, one can keep UD walls pinned when up domains are expanded, and keep DU walls pinned when down domains are expanded.

In Fig. 1(b) it is schematically shown how this can lead to directional motion of multiple domain walls in a strip with block-shaped anisotropy profile. In this device, every transition from low to high anisotropy, forms a pinning site. The first strip shows an initial magnetic configuration, which we want to shift coherently to the right through the strip. In the first step, a positive *H*_*z*_ is applied, which expands the up domains, while +*H*_*x*_ is applied such that DU walls are pinned and UD walls are free to move as long as the fields are applied. The configuration after this first step is shown in the second wire in Fig. 1(b). The second step is the application of a negative *H*_*z*_ field, which expands down domains while −*H*_*x*_ changes the DW energy landscape such that only the UD walls are pinned. This results in the configuration which is shown in the last wire, in which both domain walls have shifted to the right with respect to their initial configuration.

### Proof of principle

**Figure 2:**
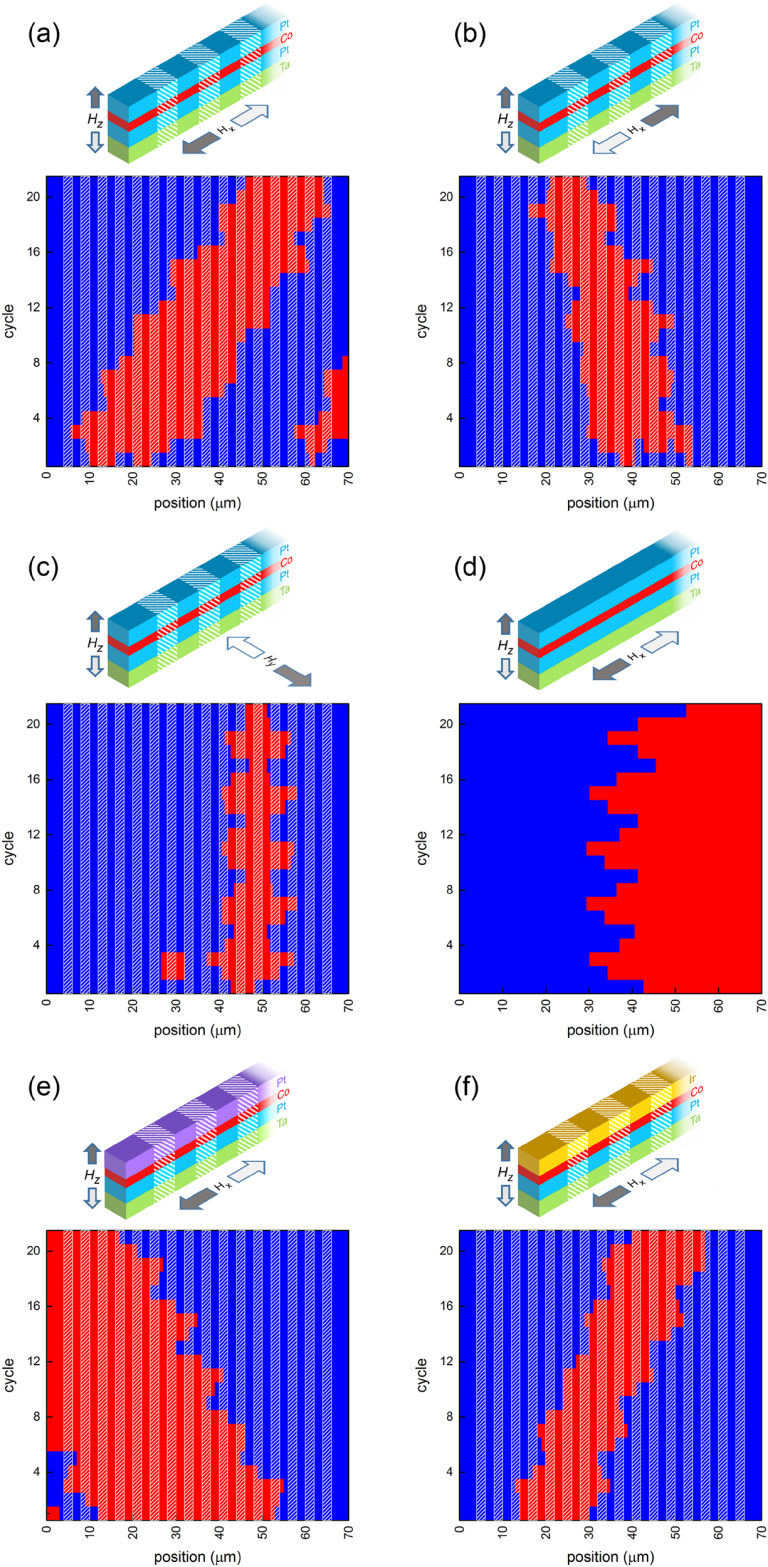
Magnetic configuration (red = down, blue = up) of a strip for every cycle in the propagation sequence. (**a**) Domain walls successfully being moved to the right, fields strengths *H*_*x*_ and *H*_*z*_ are 140 mT and 10.4 mT respectively. (**b**) Domain walls successfully being moved to the right by changing the sign of combined fields, field strengths *H*_*x*_ and *H*_*z*_ are 140 mT and 9.2 mT respectively). (**c**) In-plane fields (120 mT) are applied transverse to instead of along the strips, *H*_*z*_ = 10.8 mT. (**d**) Unirradiated strip, field strengths *H*_*x*_ = 150 mT and *H*_*z*_ = 5.0 mT are used. (**e**) Sample with top Pt layer grown under higher argon pressure, field strengths *H*_*x*_ = 80 mT and *H*_*z*_ = 22.8 mT are used. (**f**) Sample with top Pt layer replaced by Ir, field strengths *H*_*x*_ = 160 mT and *H*_*z*_ = 8.0 mT are used.

To experimentally test this device concept, 70 *μ*m long, 1 *μ*m wide Ta(5 nm)/Pt(4 nm)/Co(0.6 nm)/Pt(4 nm) strips with multiple 2 *μ*m long regions of reduced anisotropy are produced. Reducing the anisotropy locally is achieved by irradiation by a focussed ion beam, and for this particular sample a dose of 0.5 *μ*C/cm^2^ was used. During the measurements, the strips are first saturated with a positive *H*_*z*_ field, and by applying a short negative *H*_*z*_ pulse, some randomly located inverted domains are created. Now the steps as described in the previous section are performed. Experimentally, we apply a 0.5 ms *H*_*z*_ pulse together with a constant in-plane field. It was decided to always start with pulses in the −z direction, to carry out each step twice and to repeat the complete procedure five times. See Supporting information 1 for details on how the magnetic configuration of a strip is extracted from the raw experimental data (e.g. it is discussed how up and down domains can be identified automatically and how a specific domain wall is traced through subsequent images). The magnetic state of a typical strip during each cycle is shown in Fig. 2(a). The situation after the nucleation of random domains is shown in cycle 1, where the blue and red regions represent up and down domains, respectively. The vertical shaded stripes indicate the regions along the strip with lower magnetic anisotropy. The magnetic configuration after each field pulse (in this measurement −*H*_*z*_ are combined with +*H*_*x*_ fields) is shown. By reading the figure from bottom to top it can be seen how the magnetic domains move in time, and in general it can be seen that both UD and DU walls are successfully moved to the right. To complete the proof-of-principle, it is shown that coherent shift to the left is possible as well. Experimentally this is done by now combining positive *H*_*x*_ fields with positive *H*_*z*_ fields, and the results are shown in Fig. 2(b).

In order to verify our interpretation of the observation, two additional experiments were performed. Figure 2(c) shows the same experiment, but now using in-plane fields transverse to the strips instead of parallel to the strips. No unidirectional motion was observed, excluding unintentional asymmetry created during sample fabrication. Instead, it nicely corresponds to the interpretation based on DMI, where a transverse field is not expected to create a difference between UD and DU domain walls. A second control experiment was performed, in which an unirradiated sample is investigated. For typical *H*_*z*_ fields that were used before (~10 mT), the domain walls are now propagated over such a large distance that they reach the end of the strip within one field step. We repeated the experiment with a factor two smaller *H*_*z*_ fields, of which the results are shown in Fig. 2(d). Interestingly, we do not observe an asymmetric domain wall velocity, which is in agreement with earlier observations^17^. This shows that unidirectional motion shown in (a) and (b) is created by asymmetric depinning and not by asymmetric domain wall velocity, as is the case in the bubblecade memory^14^.

### Device optimization

**Figure 3:**
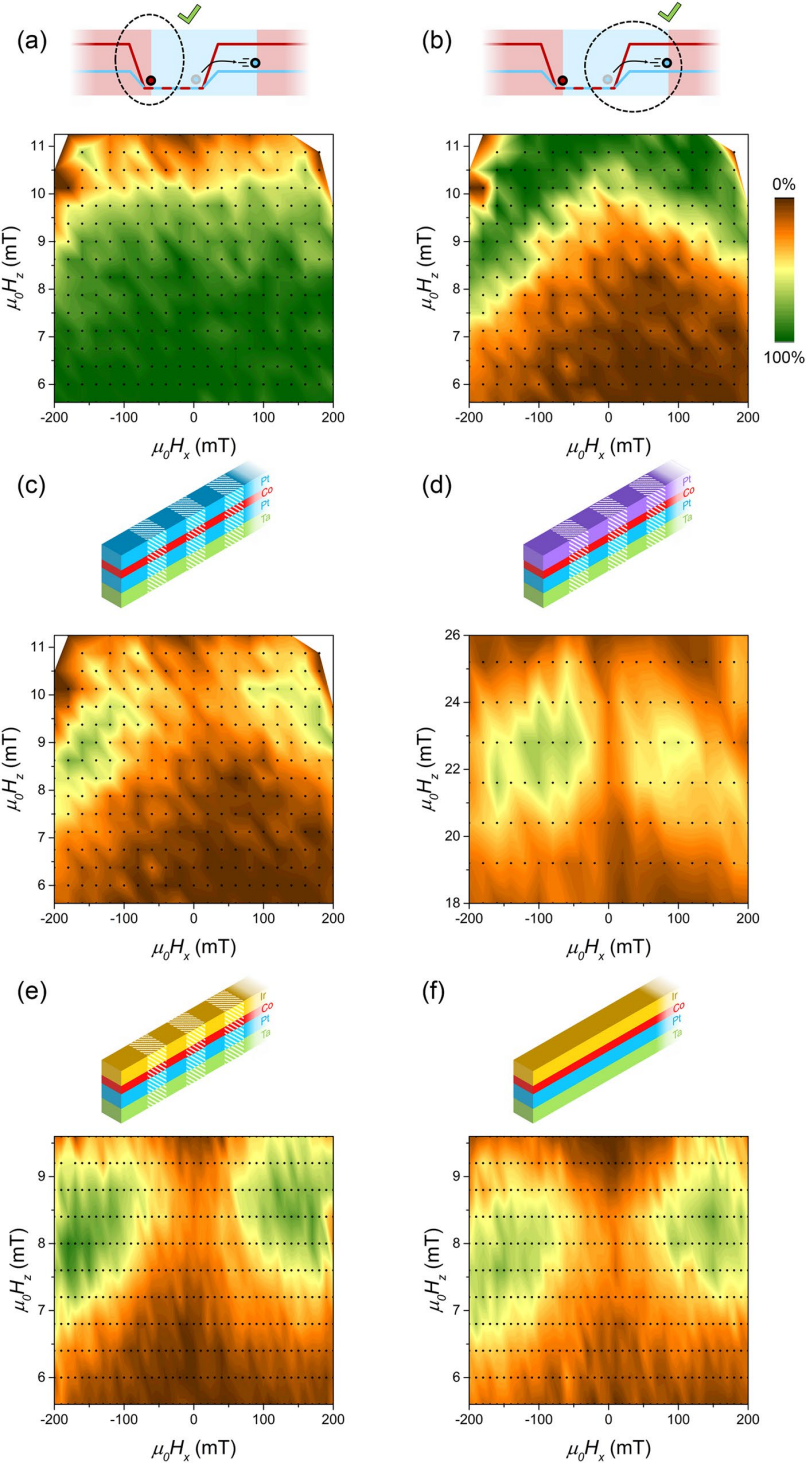
Success rate (indicated by colour scale) as a function of *H*_*x*_ and *H*_*z*_. (**a**) Chance on a successful pinning step. (**b**) Chance on a successful depinning step. (**c**) Chance that a complete procedure (both pinning and moving) is successful for the proof-of-principle sample. (**d**) Chance that a complete procedure is successful for a sample with the top Pt layer grown at a higher pressure. (**e**) Chance that a complete procedure is successful for an irradiated Pt/Co/Ir sample. (**f**) Chance that a complete procedure is successful for an unirradiated Pt/Co/Ir sample.

In Fig. 2 it can be seen that sometimes a domain wall that is supposed to propagate remains pinned, and vice versa. It is of importance to optimize the *H*_*x*_ and *H*_*z*_ field strengths that are used during the procedure to achieve a minimal amount of errors. Figures 3(a–c) show the measured success rate for the device that was used for the proof-of-principle measurements. To obtain these percentages, measurements have been performed on five strips simultaneously. The center-to-center distance of these strips is 5 *μ*m, which we have calculated to be sufficient to make dipolar fields emanating from neighbouring strips negligibly small. For each combination of field strengths, the domain walls are propagated 20 times. Though is is not possible to give an exact number because for each measurement a different number of domain walls is randomly nucleated, each percentage shown here is based on approximately 200 events. Figure 3(a) shows the measured chance that a domain wall remains pinned when it is supposed to remain pinned, which is high for small fields. Figure 3(b) shows the measured chance that a domain wall moves when it is supposed to move, which is high when large fields are used. To shift the domains coherently, it is required that both the pinning and moving step are successful. This chance is computed by the multiplication of the pinning chance with the moving chance, and the result is shown in Fig. 3(c). The plot shows two green regions with success rates of 60%. Note that the existence of these regions is not trivial; if the UD and DU depinning fields are not influenced differently, the pinning chance equals one minus the moving chance, and the product can never exceed 25%.

To ensure that the physics is ruled predominantly by DMI, it is investigated how the behaviour of the device changes when the DMI is increased. Because this increases the asymmetry between UD and DU walls, this is expected to improve the reliability of the proposed field-driven racetrack as the region of high total success rate should be expanded. We have investigated two alternative sample stacks: (i) a sample for which the top Pt layer is grown at a different pressure and (ii) a sample for which the top Pt layer is replaced by an Ir layer. These stacks are chosen because DMI is affected by both interface engineering by altering the growth kinetics^17^ and variation of the used materials at the interfaces^23^. Because these new stacks are structurally less symmetrical than the Pt/Co/Pt sample that was used up to now, the DMI (and therefore the success rate of the racetrack) is expected to be increased. The DMI was not measured independently for the samples used in this work, but studies of similar samples, grown in the same lab, can be found in literature^17, 24^.

A typical result for a sample with a Pt top layer grown at 1.12 Pa instead of 0.29 Pa is shown in Fig. 2(e). Figure 3(d) shows the success rate at various field combinations. The maximum success rate is comparable to the one obtained for the standard Pt/Co/Pt sample, though is reached at significantly higher *H*_*z*_ fields. The most striking difference can be seen when comparing Fig. 2(a) to Fig. 2(e): while the same field polarities are used, the direction of the domain wall motion is surprisingly opposite! For Pt/Co/Pt samples both observations of a dominant DMI contribution from the bottom interface as from the top interface have been reported^16, 23, 25^, suggesting a sensitive dependence on the interface quality. When the growth pressure is increased the growth kinetics, and therefore the interface quality, changes. It was observed that increasing the growth pressure for the top Pt layer drastically increases the magnetic surface anisotropy (which also explains why a larger difference in anisotropy could be created and higher *H*_*z*_ fields were required), which suggest that the interface quality is improved. Therefore we speculate that the change in direction of domain-wall motion might be due to a sign change in effective DMI by a change in the dominant interface, which would be a surprisingly large effect of such a subtle modification of the material stack. Support for this theory is found in one of our ongoing projects in which the equilibrium DW configuration in magnetic strips under the influence of in-plane fields is investigated. Also in these experiments opposite results are found for the same material stacks, again explicable by a opposite sign of the DMI^26^. However, because also the irradiated regions play a role in our racetracks, caution should be taken with this conclusion. It has been reported that ion irradiation affects the structural properties of the top and bottom interfaces in a Pt/Co/Pt sample differently^27^, so it is not unlikely that the DMI is affected.

Large effective DMI strenghts have been observed for Pt/Co/Ir stacks^24, 28^. In contrast to symmetric stacks where the interfacial DMI of both interfaces (partially) cancel each other, this system could have an enhanced total DMI, because opposite signs of the DMI are expected for Pt/Co and Ir/Co interfaces^23, 29, 30^ (though recently conflicting observations were reported^31^). We conclude our study on the field-driven racestrack concept with measurements on a Ta(5 nm)/Pt(4 nm)/ Co(0.8 nm)/Ir(4 nm) sample. Figure 3(e) shows the success rate at various field combinations for strips irradiated with a dose of 0.2 *μ*C/cm^2^. Large regions with a success rate of 80–90 percent are observed, a clear and significant improvement with respect to the Pt/Co/Pt samples, underlining the importance of DMI for these devices. The choice for the low irradiation dose used on this sample is necessary because for the slightly higher dose of 0.4 *μ*C/cm^2^ the nucleation field in the irradiated regions drops below the depinning fields. This triggered us to repeat the test of a device without irradiation for this sample. Surprisingly, the results, shown in Fig. 3(f), are completely different from the unirradiated Pt/Co/Pt sample. In Fig. 2(d), though this shows a single measurement instead of a phase diagram, it can be seen that there is no unidirectional domain-wall motion for the Pt/Co/Pt sample. For the Pt/Co/Ir sample however, the success rate is considerable, and the maximum occurs at similar fields as for the irradiated Pt/Co/Ir sample. As no anisotropy profile is created in this sample, the unidirectional displacement must be due to an asymmetry in DW velocity, similar to the principle behind the bubblecade memory^14^. This means that when analyzing the irradiated Pt/Co/Ir sample two DMI related phenomena have to be considered: both the asymmetry in DW velocity and in depinning field play a role, making this particular device very interesting.

**Figure 4:**
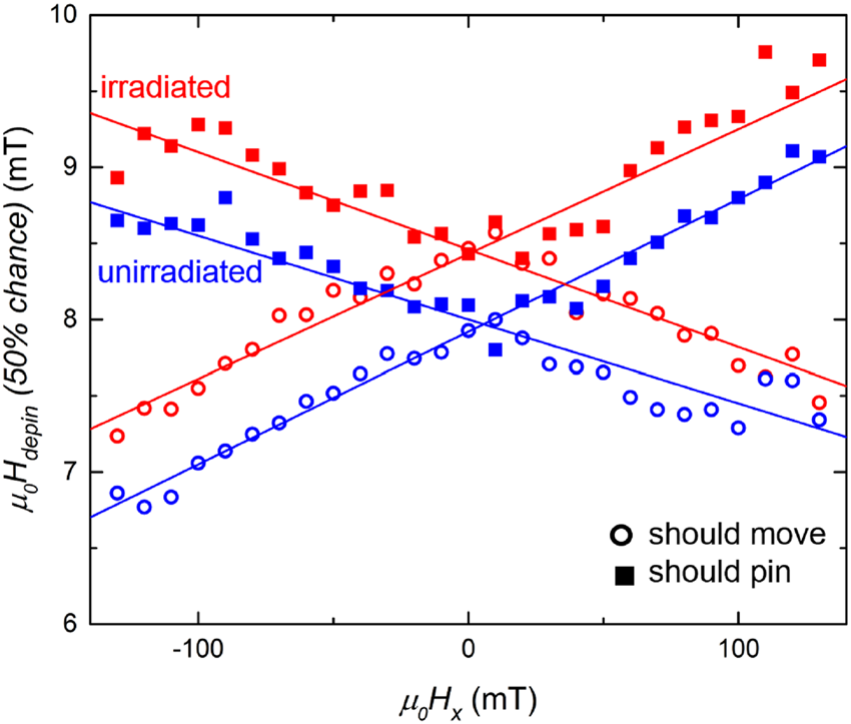
Boundaries for which a domain wall has 50 percent chance to depin, for both the irradiated and unirradiated Pt/Co/Ir sample and for both domain walls that are suppose to remain pinned and for domain walls that are supposed to move.

In order to unravel the physical origin behind these contributions, we further investigate the difference between the irradiated and unirradiated Pt/Co/Ir sample. First it is verified that domain-wall motion follows the creep law in both irradiated and unirradiated samples, and that the domain-wall velocity is not significantly altered by irradiation, see Supporting information 2. Figure 4 shows boundaries at which the chance of moving and staying pinned are 50 percent, both for domain walls that are supposed to move and supposed to pin. The curves for the irradiated sample are clearly shifted towards higher *H*_*z*_ fields by an amount of 0.49 ± 0.06 mT. This makes sense; when barriers are introduced it will be less probable to propagate than when there are no barriers. Interestingly the slopes of the graphs are identical within the margin of error for the irradiated and unirradiated sample. This seems to indicate that apparently no additional asymmetry with in-plane field is created by the irradiation, in striking contrast to the Pt/Co/Pt case. An explanation could be that the effect is simply very small at this small irradiation dose. However, the irradiation seems to have a positive effect on the performance of the device, as the maximum success rate is clearly higher for the irradiated sample. In-depth analysis of the data, see Supporting Information 2, shows that the transition between the situation in which a domain wall moves or pins becomes more abrupt after irradiation. The reason for this is that without irradiation only the chance that a domain wall reaches the barrier position plays a role, but for the irradiated sample this chance has to be combined with the chance that the domain wall can depin from this barrier. When the field range for which only one type of wall can move past a barrier stays equally large, this more abrupt transition leads to a higher maximum success rate.

## Discussion

The results of the device optimization lead to an unexpected conclusion: by varying the material stack, we have created two types of devices, both of them function, but have a different underlying principle. The Pt/Co/Pt devices are based on a difference in depinning field for UD and DU walls, as explained before. The Pt/Co/Ir samples, however, get their unidirectionality from a difference in velocity between UD and DU walls, similar to the principle behind the bubblecade memory, but now using domain-walls instead of magnetic bubbles. The Pt/Co/Ir sample has the highest success rate of the devices investigated so far. This does not automatically mean that the difference in velocity is the best basis for a field-driven racetrack. When material stacks with larger differences in depinning field for UD and DU walls are designed, also a larger success rates are expected. Therefore both types of devices are interesting candidates for future memory applications.

The size of the regions with reduced anisotropy in the investigated samples was 2 *μ*m. This choice was made to enable the Kerr microscope imaging, but does not reflect the fundamental limits of the device. One may wonder whether the use of a focussed ion beam does not significantly increase the minimal bit size, which is, however, not the case. First, the width of the created anisotropy boundary is 22 nm, but could be further reduced by switching from Ga ions to, for instance, He ions^32^. Secondly, the actual limiting factor is the minimal distance between two domain walls at which they do not interact with each other by dipolar fringe fields, which is considerably more than 22 nm^33, 34^, just as in a traditional racetrack device. Finally, we note that for the simple anisotropy profile we need to create, a *focussed* ion beam is not essentially required. The ion irradiation could be done through a mask^35^, greatly reducing the sample fabrication time.

Another concern could be the domain wall velocity that poses a fundamental limit to the operation speed of the device. This velocity is related to *H*_*z*_ which unfortunately cannot be increased arbitrarily, because it is required to be in a specific range in order to make the device function, see Fig. 1(a). For the samples investigated, this is in the creep regime and the velocities are in the order of 10^−3^ m/s. To improve the speed, a different material stack and irradiation dose, resulting in a higher depinning field, could be used. Moreover, the theoretically possibility to have this type of racetrack operating with higher domain wall velocities (and smaller sample dimensions) is demonstrated by micromagnetic simulations, see Supporting Information 3.

An advantage of the proposed device over conventional current driven racetracks is the creation of discrete positions at which the domain wall can be located (how it can be achieved that the domain walls certainly end up at these anisotropy barriers is discussed in Supporting Information 3). In studies on current induced domain wall motion, asymmetric domain wall depinning in the presence of in-plane fields was also observed^36, 37^. This implies that the advantageous pinning sites tunable by in-plane fields that were investigated in this work, could be extended to current-driven devices.

Though the development towards devices that are of interest for industry should be possible in theory, as discussed above, this will certainly not be trivial. An example of a technical issue is the quality of the strip edges. These will have to be very smooth for nanoscale devices to prevent them from forming pinning sites that dominate over the pinning by barriers induced by irradiation, which makes the lithography challenging. Another example is making a sample design in which magnetic fields can be applied locally, which is necessary in applications where it is desirable to control the domain walls of only one strip situated within a large array of strips. However, these issues are beyond the scope of this work.

Finally, the rather elementary devices shown in this manuscript are purely meant to demonstrate the proof-of-principle of this DMI-based racetrack memory, and therefore have the geometry of simple strips. However, extension to a second dimension can open up new possibilities: domain walls could be selectively moved through a grid by using magnetic fields in the *y* direction as well, or operators for domain wall logic could be designed using this concept of domain wall motion.

In summary, we have presented a technique to achieve unidirectional domain wall motion based on the chiral dependence of the depinning field on in-plane fields. Proof-of-principle measurements were shown and a number of material stacks were investigated to explore the possibilities and requirements of this novel device.

## Methods

Both Ta(5.0 nm)/Pt(4.0 nm)/Co(0.6 nm)/Pt(4.0 nm) and Ta(4.0 nm)/Pt(4.0 nm)/Co(0.8 nm)/Ir(4.0 nm) were deposited on a Si O_2_ (100 nm) substrate in a UHV magnetron sputtering facility. The 1 *μ*m × 70 *μ*m strips were created by standard electron-beam lithography and lift-off techniques. The 2 *μ*m long regions of lower magnetic anisotropy were created by focussed- ion-beam irradiation using a FEI Nova Nanolab 600 dualbeam system. All measurements are performed using an Evico Kerr microscope and home-built electro magnets.

## Electronic supplementary material

Supplementary information racetrack memory based on in-plane-field controlled domain-wall pinning
