# Supplementary material for: Racetrack memory based on in-plane-field controlled domain-wall pinning
Source: Sci Rep. 2017 Apr 11;7:833. doi: 10.1038/s41598-017-00837-x (PMC5429776; doi:10.1038/s41598-017-00837-x)
Supplement: Supplementary file 1 — Supplementary information racetrack memory based on in-plane-field controlled domain-wall pinning [file 41598_2017_837_MOESM1_ESM.pdf]

Racetrack memory based on in-plane-field controlled domain-wall pinning

Fanny Ummelen, Henk Swagten, and Bert Koopmans

S1. ANALYSIS PROCEDURE

Converting the Kerr microscope data in the presented experiments to domain wall position is not trivial. In this section we will discuss this analysis procedure in detail.

The Kerr intensity is measured along the 70 μm long, 1 μm wide strips by dividing them into 70 adjacent 1 μm long (or 100 adjacent 0.7 μm long) regions of interest (ROI) (for smaller regions of interest the signal to noise ratio becomes unworkable small), see figure S1 (a). Now it has to be determined in which ROIs the magnetization is up and in which it is down. Due to the random creation of domains during the nucleation step, this cannot be done by simply checking whether the Kerr intensity is above or below the average value. The up and down domains are not necessarily equal in size, or the strip may even be entirely up (or down). To solve this, a histogram is made of the intensity measured in the different ROIs. This is fitted with a mixture of two normal distributions, see figure S1 (b). If the difference in the central value of the two normals exceeds a certain empirically chosen value, and both normals contribute at least 3 percent to the total fit function, we conclude that both up and down domains are present. Whether a certain ROI is up or down is determined by checking whether its intensity is above or below the average of the two normal central values.

The domain wall positions along the strip after every field step have now been identified. However, it is not trivial which domain walls observed at different steps correspond to each other. We cannot simply say that the most left domain wall after the nucleation step is the same as the most left domain wall after the first propagation step. This is because domain walls can disappear when they reach the end of the strip, domain walls can annihilate when an ud and an du wall meet, and new domains can be nucleated if large H_z fields are used. In our analysis, we start with a domain wall in the initial configuration, and then assume after a cycle that the domain wall that is closest by and of the same type (an ud wall cannot be changed to an du wall) is that same domain wall. This solves the mentioned problems in most cases, as shown in figure S1 (c), but can still lead to a some errors, especially when large magnetic fields are used.

Now we have data on how the individual DWs move through strip in time. To analyse the performance of the ratchet, we need to know whether a DW stays pinned when it is supposed to and whether it is propagated when it is supposed to. The location of the

anisotropy barriers is determined during sample fabrication and is known. For every step, it is checked what is the closest barrier to the DW, and in the next step it is checked whether the domain wall has past this barrier or not. There is no additional check whether a DW passes one barrier or more. This is no problem at the moment, but should be addressed in a later stage of the development of the device. If multiple barriers are passed within one cycle the device still functions, but the space required for one bit of information increases, which is undesirable for data storage applications.

Last, the Kerr microscope measurements take several hours, so a small drift in the sample position can occur. Therefore the first and last ROI sometimes come to cover not only the magnetic strip, but also (partially) the substrate, for which the measured intensity is completely different. This can result in the detection of a 'domain wall' that stays at the same position throughout all cycles, which changes the measured pinning/depinning probability. Therefore the first and last ROI are not included in the analysis, which is no problem as the created anisotropy profile does not extend to there.

S2. INFLUENCE OF IRRADIATION ON PT/CO/IR

In the main manuscript it was observed that a Pt/Co/Ir strip could show directional domain wall motion using our propagation scheme, even without anisotropy barriers created by ion irradiation. In this part we further investigate the influence of additional ion irradiation on the functionality of a device consisting of this material stack.

One difference between the Pt/Co/Pt and Pt/Co/Ir samples is the thickness of the Co layer; for Pt/Co/Pt a thickness of 0.6 nm was used, while for Pt/Co/Ir a thickness of 0.8 nm was used. The choice for 0.6 nm in Pt/Co/Pt was a practical one, for this particular material stack measurements of the anisotropy and dose tests for ion irradiation were available. On the choice for 0.8 nm in Pt/Co/Ir, we will elaborate here, as the behaviour of this material stack is central in this section. The main reason for the study of Pt/Co/Ir is to see how the devices behave if the DMI is large. Because the DMI in these samples is an interfacial effect, its energy contribution per area of a domain wall becomes larger for thinner layers, so making the Co layer as thin as possible would be preferable. It turns out that, for samples grown in our sputtering facility, the range of thicknesses for which a sample shows perpendicular magnetic anisotropy (PMA) is different for Pt/Co/Pt and Pt/Co/Ir,

for Pt/Co/Ir the minimum Co thickness to observe PMA is larger. We speculate that this is related to the formation of a magnetically dead layer. Within the range were Pt/Co/Ir shows PMA, the smallest Co thickness is chosen, but at a safe distance from the transition where the PMA disappears. This safe distance makes it possible to still have PMA after ion irradiation.

Domain wall motion in Pt/Co/Pt films grown in our sputtering facilities was extensively studied before [1], whereas in Pt/Co/Ir it was not. Therefore we start with domain wall velocity measurements as a function of H_z in absence of any in-plane field. Both a pristine strip and a homogenously irradiated strip (dose 0.2 μC/cm^2) were investigated. In figure S2 the natural logarithm of the measured velocity is plotted against $H_z^{-1/4}$. This results in a straight line (for both the irradiated sample, figure (b), and the pristine sample, figure (a)) indicating that the domain wall motion follows the creep law $v = v_0 \exp\left(-\chi(\mu_0 H_z)^{-1/4}\right)$, just as in the Pt/Co/Pt measurements. In this equation v_0 is the characteristic speed and χ is a scaling constant. Interestingly, there is no significant difference between the fit parameters obtained for the pristine sample ($\chi = -5.6 \pm 0.2$, $\ln v_0 = 13.4 \pm 0.5$) and the homogeneously irradiated sample ($\chi = -5.6 \pm 0.3$, $\ln v_0 = 13 \pm 1$), implying that the domain wall motion is not affected by the irradiation.

Next we further analyse the data shown in figure 3 (e) and (f) of the main manuscript by looking at the pinning and propagation chances separately instead of at the total success chance. Figure S2 (c) shows the chance that a domain wall moves over the closest barrier when it is supposed to. Figure S2 (d) shows the chance that chance that a domain wall does not move over the barrier when it is supposed to remain pinned. Data for both an unirradiated sample and a sample with a block shaped anisotropy profile created by irradiation are shown. For the unirradiated strips we can formulate an expectation for the pinning chance as a function of H_z. Because there are no barriers, the success chance is computed by checking whether the domain wall passes the point where there would be a barrier in an irradiated sample. We assume that the distance between a domain wall and the nearest barrier it has to pass is a random distance between 0 and 4 μm (because the distance between two neighbouring barriers is 4 μm). The used pulse duration is 500 μs (though the effective time will be a bit less, because of the finite rise time), so the measured propagation chance should be proportional to the DW velocity (which dependence on H_z is given by the creep law), until a velocity is reached for which more than 4 μm is travelled during one pulse. Fits

using this model are shown in figure S2 (c) and (d) as the black curves.

For the sample in which an block shaped anisotropy profile is created by irradiation (blue points), this model describes the data less accurately (fit is not shown). This is because besides the chance that the domain wall reaches the barrier, now there is also a chance that the domain wall cannot overcome the barrier, which is not yet taken into account. The model is now improved in the following way: we assume that the chance, p, that the anisotropy barrier is overcome follows an Arrhenius law, $p = p_0 \exp(-\frac{E}{k_b T})$ [2]. Now the chance that a domain wall moves past the barrier is a product of the chance that the barrier is reached and and the chance that the barrier is overcome. For the first contribution, the χ and $\ln v_0$ from the unirradiated sample can be used, because the study of the homogeneous irradiated strip showed that the irradiation did not chance these values significantly. For the chance that the barrier is overcome, p_0 (a parameter related to the attempt frequency) and the energy barrier E are left as fit parameters. Examples of fits with this product of two chances is shown in figure S2 (c) and (d) as the blue curve. This improved model now turns out to be able to describe the measured points nicely.

Please note that the analysis presented here is specific for the case in which the initial positions of the domain walls are random. This is indeed the case in the experiments presented in the main manuscript, but one could envision that in future devices it would be desirable to controllably write domains at a specific location. In that case, the chance that a domain-wall reaches a pinning barrier is no longer given by $(velocity \times pulse\, time)/4\,\mu\text{m} \times 100\%$ and the analysis would have to be reworked. However, as this is not applicable for the experiments shown in this work, an alternative analysis is beyond the scope of this work and will not be given here.

When comparing the data for the unirradiated and the irradiated samples in figure (c) and (d) two differences stand out. First, the H_z field at which a domain wall moves (or fails to pin) is shifted towards higher values for the irradiated samples. This is expected, because the barriers introduced by the irradiation will make it more difficult for the domain walls to move and it is in agreement with figure 4 in the main manuscript. The other difference is that the transition from pinning to moving is less gradual in the irradiated sample. This makes it possible to reach a higher total success rate, which explains the difference between figure 3 (e) and (f). In conclusion, the irradiation on the Pt/Co/Ir does not create an additional asymmetry with in-plane field due to an asymmetry in the depinning field, but it

influences the success rate by making the transition from pinning to moving more abrupt.

S3. OOMMF RESULTS

One advantage of our device concept is that the domain walls can be located at discrete positions, namely the anisotropy barriers, but this is not trivial. DWs that are pinned end up at a barrier, but the ones that are moved can end up at any position, depending on the duration of the field pulse. This problem can be solved by pulsing the H_x instead of the H_z field. This way the domain walls can only depin during the short H_x pulse, but they will keep moving (driven by H_z) until they reach the next anisotropy barrier. Using our setup this would be a tedious experiment. The coil used to generate the H_z field is integrated in the sample stage. If this field is not pulsed but turned on for a longer time, heating causes the sample stage to expand and bring the sample out of the focus of the microscope. So instead we investigate this alternative scheme by object oriented micro magnetic framework (OOMMF) simulations [3]. Also, OOMMF simulations enable us to study the DW motion in the absence of random pinning sites or thermal fluctuations. This means that the domain wall motion in the simulations is in the flow regime per definition, allowing us to investigate the device concept also in this regime. Last, the simulations make it possible to study the device on time and length scales that are not accessible in our proof-of-principle experimental setup.

The cellsize in the simulations is chosen to be 4 x 4 nm; simulations with larger cellsizes would be unreliable because the cell size would then exceed the typical domain wall width. The geometry is a 2100 nm long, 156 nm wide and 1 nm thick strip, with regions of lower anisotropy that are 100 nm wide and 100 nm separated from each other. This is considerably smaller than the experimental strips (70 μm long) and this decided both to reduce computing time and to explore these small dimensions that are more interesting for applications. The material parameters used are $M_\mathrm{S} = 1.4 \cdot 10^6$A/m (bulk value for Co), $K = 1.5 \cdot 10^6$ J/m^3, $A = 1.6 \cdot 10^{-11}$ J/m, $D = 0.2$ mJ/m^2 and in the irradiated regions K and D are reduced by 10% and 100% respectively. In preparatory simulations, in which the depinning field was determined, also other parameters were investigated. The anisotropy in in the irradiated area was reduced by 5, 10, 15 and 20 percent. The DMI constant of the pristine region was set to 0 mJ/m^2, 0.2 mJ/m^2 or 0.5 mJ/m^2, which are moderate value in order to mimic our

symmetric Pt/Co/Pt stack. Reductions of the DMI of 0, 15 and 100 percent in the irradiated region were tried. Which of these is most realistic is unclear at this moment, we are still awaiting results of Brillioun light scattering measurements to clear up this point. In almost all cases an difference between the depinning field of UD and DU walls could be created using an in-plane magnetic field, the exceptions are simulations in which there is no DMI in both the pristine and irradiated regions. We take one typical combination of parameters to show that this difference can be used to create unidirectional domain wall motion in the simulation. To investigate a large parameter space and create a phase diagram is beyond the scope of this paper.

During one propagation step a magnetic field $H_z = 45$ mT is applied for 7 ns, during which a magnetic field pulse $H_x = 50$ mT is applied for 2 ns. Figure S3 shows the evolution of the magnetic domain structure together with the anisotropy profile. The domain wall motion is 100% reliable in these pinning and noise free simulations. Also note that the domain walls now always end up at the anisotropy transitions, as is desired. This result shows the working principle of the device using H_x pulses, and shows the theoretical possibility of operating these devices in the flow regime of domain wall motion.

---

[1] R. Lavrijsen, D. M. F. Hartmann, A. van den Brink, Y. Yin, B. Barcones, R. A. Duine, M. A. Verheijen, H. J. M. Swagten, and B. Koopmans, Phys. Rev. B **91**, 104414 (2015).

[2] C. Burrowes, A. Mihai, D. Ravelosona, J.-V. Kim, C. Chappert, L. Vila, A. Marty, Y. Samson, F. Garcia-Sanchez, L. Buda-Prejbeanu, I. Tudosa, E. Fullerton, and J.-P. Attané, Nat. Phys. **6**, 17 (2010).

[3] M. Donahue and D. Porter, *OOMMF User's Guide Version 1.0*, Tech. Rep. (National Institute of Standards and Technology, Gaithersburg, MD, 1999).

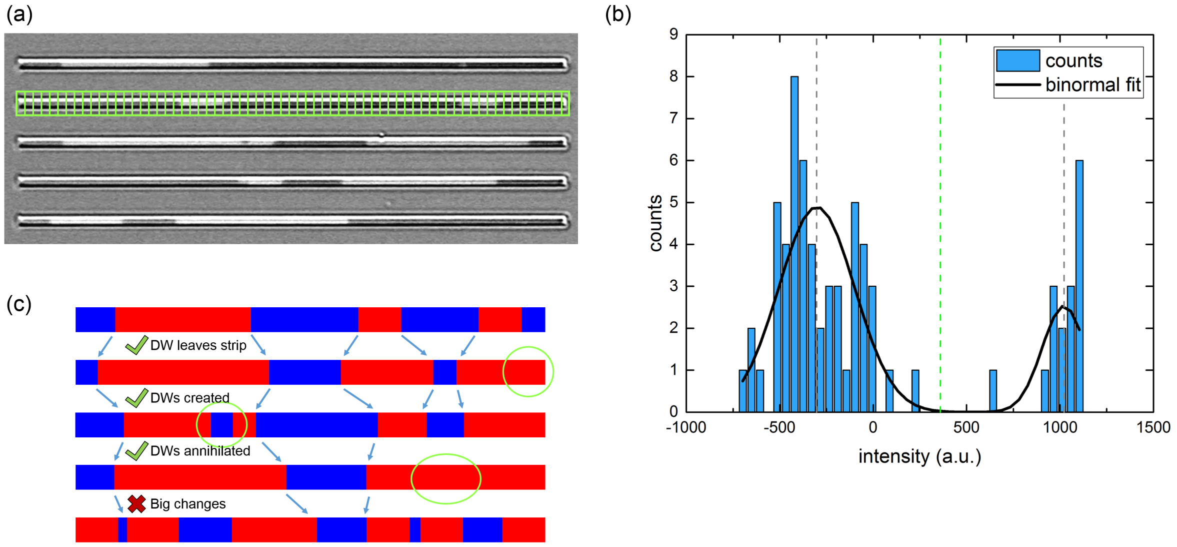

FIG. S1. Extracting domain-wall position and displacement from Kerr microscope images. (a) Typical Kerr microscope image of 5 devices with several domain walls. The strips are divided into small regions of interest (ROI), typically 1.0 μm or 0.7 μm wide, as is schematically shown for the second strip. For each ROI the intensity is measured and a histogram can be made of how often certain ranges of intensities occur (b). This is fitted with a mix of two normal distributions such that the average values corresponding to up and down domains (grey dashed lines) can be identified. The mean of these two (green dashed line) can be used to select which ROIs are identified as up and down. (c) Example of how the software follows DWs through different cycles. In most cases, creation and annihilation of DWs or DWs moving to the edge of the strip do not cause problems. When too large fields are used and many of these events occur within one cycle the process becomes unreliable.

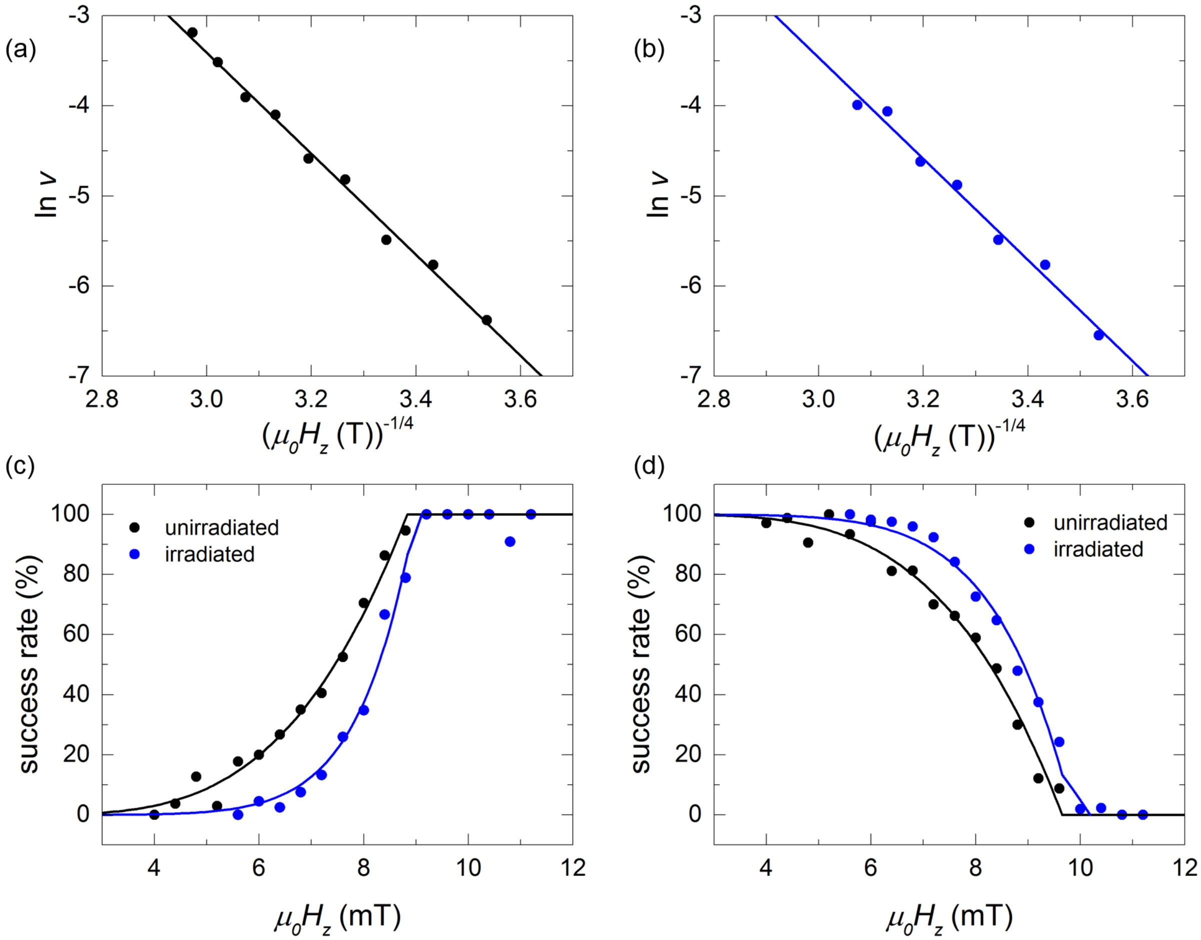

FIG. S2. Investigation of the influence of irradiation on Pt/Co/Ir samples. Velocity versus H_z measurement of an (a) unirradiated Pt/Co/Ir strip (b) homogeneously irradiated Pt/Co/Ir strip using a dose of 0.2 μC/cm^2. Points indicate the measured values, the line shows a linear fit. Chance that a domain wall (c) moves when it is supposed to move (d) stays pinned when it is supposed to stay pinned as a function of H_z, with an inplane field strength of 50 mT. The black points are measurements on an unirradiated strip, the blue on a strip with an anisotropy profile created by irradiation. Curves are fits using the model described in the text.

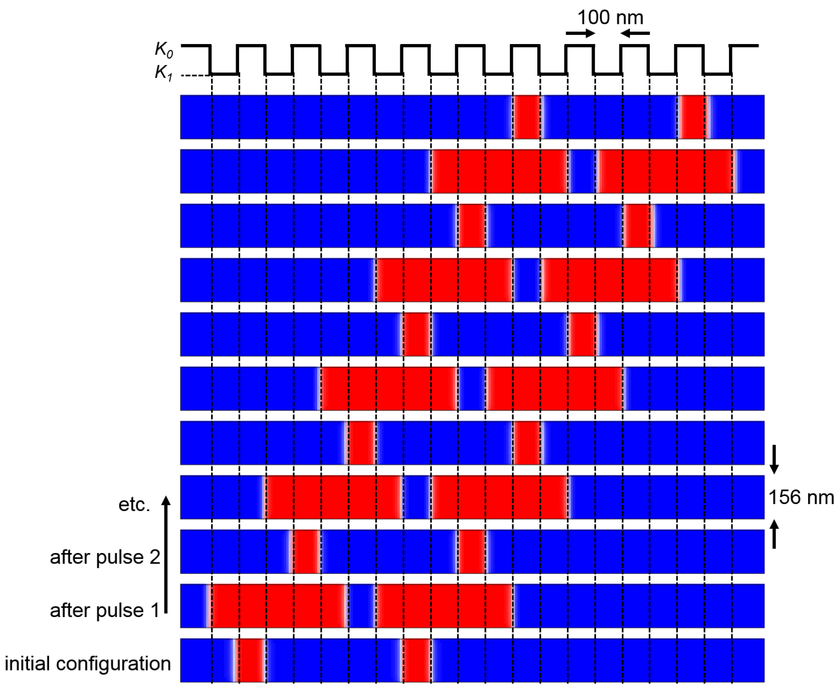

FIG. S3. Magnetic configurations obtained by OOMMF simulations. The bottom picture shows the initial configuration, from bottom to top configurations after subsequent field pulses are shown. The schematic block profile shows the anisotropy variation along the strip.
